# Retraction: Monodispersed palladium–cobalt alloy nanoparticles assembled on poly(*N*-vinyl-pyrrolidone) (PVP) as a highly effective catalyst for dimethylamine borane (DMAB) dehydrocoupling

**DOI:** 10.1039/d1ra90154k

**Published:** 2021-10-12

**Authors:** Laura Fisher

**Affiliations:** Royal Society of Chemistry, Thomas Graham House Science Park, Milton Road Cambridge UK advances-rsc@rsc.org

## Abstract

Retraction of ‘Monodispersed palladium–cobalt alloy nanoparticles assembled on poly(*N*-vinyl-pyrrolidone) (PVP) as a highly effective catalyst for dimethylamine borane (DMAB) dehydrocoupling’ by Betül Çelik *et al.*, *RSC Adv.*, 2016, **6**, 24097–24102, DOI: 10.1039/c6ra00536e.

The Royal Society of Chemistry hereby wholly retracts this *RSC Advances* article due to concerns with the reliability of the data in the published article.

The low angle portions of the three XRD spectra in Fig. 2, representing three different materials: Pd@PVP, Co@PVP and Pd–Co@PVP NPs, are the same. The blue spectrum representing Co@PVP also has repeating fragments at higher angles. An expert reviewed the authors’ responses but concluded that they did not satisfactorily address the concerns, and that the replacement data provided by the authors did not fully support the conclusions. Given the significance of the concerns about the validity of the data, the findings presented in this paper are no longer reliable.

Fatih Sen, Betül Çelik and Hakan Sert oppose this retraction. Esma Erken, Yunus Yıldız and Yagmur Koskun were contacted but did not respond.

Signed: Laura Fisher, Executive Editor, *RSC Advances*

Date: 23rd September 2021

## Supplementary Material

